# Methods for Proteogenomics Data Analysis, Challenges, and Scalability Bottlenecks: A Survey

**DOI:** 10.1109/ACCESS.2020.3047588

**Published:** 2020-12-25

**Authors:** MUHAMMAD USMAN TARIQ, MUHAMMAD HASEEB, MOHAMMED ALEDHARI, REHMA RAZZAK, REZA M. PARIZI, FAHAD SAEED

**Affiliations:** 1 School of Computing and Information Sciences, Florida International University, Miami, FL 33199, USA; 2 College of Computing and Software Engineering, Kennesaw State University, Marietta, GA 30060, USA

**Keywords:** Proteogenomics, proteomics, high-performance computing, workflow, genomics, big data, mass spectrometry

## Abstract

Big Data Proteogenomics lies at the intersection of high-throughput Mass Spectrometry (MS) based proteomics and Next Generation Sequencing based genomics. The combined and integrated analysis of these two high-throughput technologies can help discover novel proteins using genomic, and transcriptomic data. Due to the biological significance of integrated analysis, the recent past has seen an influx of proteogenomic tools that perform various tasks, including mapping proteins to the genomic data, searching experimental MS spectra against a six-frame translation genome database, and automating the process of annotating genome sequences. To date, most of such tools have not focused on scalability issues that are inherent in proteogenomic data analysis where the size of the database is much larger than a typical protein database. These state-of-the-art tools can take more than half a month to process a small-scale dataset of one million spectra against a genome of 3 GB. In this article, we provide an up-to-date review of tools that can analyze proteogenomic datasets, providing a critical analysis of the techniques’ relative merits and potential pitfalls. We also point out potential bottlenecks and recommendations that can be incorporated in the future design of these workflows to ensure scalability with the increasing size of proteogenomic data. Lastly, we make a case of how high-performance computing (HPC) solutions may be the best bet to ensure the scalability of future big data proteogenomic data analysis.

## I. INTRODUCTION

Integrating data from next generation sequencing technology (NGS) for genomics and high-throughput mass-spectrometry (MS) of proteomics has given rise to an emerging field of big data proteogenomics. Proteogenomics has a wide range of applications of crucial importance including: microbiology [1], bacteriology [2], virology [3], gene annotation [4], [5], human neurology [6], protein-coding genes [7], [8], cancer-biology [9], and countering bio-terrorism [10]. Historically, proteogenomics has been restricted to improving protein identification by searching the experimental spectra in the sample specific genomics data [11], [12]. More recently, efforts to utilize proteogenomic concepts to improve genome annotation [13], detecting peptides unique to individual genome [14], protein mapping of genome data [15], alternative open reading frame translations [13], translation start site identification, and finding evidence for translation [11] have started to appear frequently in literature. Proteogenomic analysis allows exhaustive search to discover novel peptides by searching each conceivable peptide using six-frame translation, splice graph, or other customized protein databases.

Proteogenomics requires scientists to integrate and process data from two high-throughput technologies: (1) mass spectrometry (MS) for proteomics [16]-[27], and (2) next generation sequencing (NGS) for genomics [28]-[32]. Both of these technologies can produce an enormous volume and velocity of data. For instance, NGS technologies such as Pyrosequencing [28], and Chip-Seq [33] can generate 600Gb/run and 1.8 Tera-bytes of DNA/RNA data in a single day [34]. Mass spectrometers can generate millions of spectra [35], [36] in a few hours [35] and the number of distinct peaks that needs to be analyzed is enormous (e.g. 4000 peaks per spectrum [37] and 250 million distinct peaks for 60k human proteins). Integration of these high-throughput data gives rise to the field of big data proteogenomics and existing tools are inadequate to deal with this data-deluge.

In the field of proteomics, and proteogenomics much energy is dedicated to formulating workflows that can work on the data to get the correct interpretation of the MS data, e.g., TOPP (OpenMS) [38], and Galaxy [39]. However, not much work has focused on developing techniques that could make these workflows more scalable, especially in the context of high-performance architectures and algorithms. This article aims to highlight several workflows proposed and the scalability issues associated with these algorithms when big data has to be analyzed. The previous survey works [40]-[44] include a description of methodologies, algorithms, and what are the challenges from the systems biology perspective, but do not discuss the issue of scalability. We address topics that need additional research requiring concentrated efforts of computational scientists and will lead to new research directions. Since high throughput proteogenomics requires expertise and insight in NGS based genomics and MS-based proteomics data, we review the relevant parts to proteogenomics data analysis.

The contributions of this article, relative to the recent literature in the field, can be summarized as:

- Compared to other survey papers in the field, this article provides a more in-depth summary of the relevant proteogenomic tools available for big data analysis.
- We provide an overview of some of the critical scalability bottlenecks and challenges identified for big data proteogenomic. Moreover, we explore the relation between big data and high-performance computing methodologies and what research directions are needed for further development.
- Like previous surveys, there is ample information about proteogenomic for researchers working in the field. Scientists working in the field will get excellent insights into the scalability and limitation of the existing workflows and what kind of tool would be best suitable for their data and experiments.

To reach a broader audience for this article, we will only assume a general computing knowledge for the reader. Our emphasis will be on the classification of the existing proteogenomic literature, developing a perspective on the scalability of the proteogenomic tools, evaluating current trends in algorithm designs, and providing recommendations for future developments. This work will also greatly benefit those specializing in bioinformatics, computational biology, computer science, and other disciplines by offering insight into current findings and their use to instill even better computational frameworks to improve this field further.

### A. PAPER ORGANIZATION

The structure of this article is as follows: In the next section, we describe the general way in which proteomics and proteogenomics data analysis is usually done. In section II, we explain the state-of-the-art genomics data preparation, sequencing, and assembly tools and generation of customized protein databases using different methods. In section III, we highlight the sequence technologies and the relevant material to proteogenomics. In section IV, we will discuss the specifics of mass spectrometry-based proteomics, including data-dependent acquisition, data-independent acquisition, and available tools for processing of MS data for peptide identification. In section V, we discuss the proteogenomics analysis and the available workflows, including the search engines used, their scoring mechanism, and their limitations in terms of data processing. We also survey which of the algorithmic workflow can exploit the high-performance computing architectures such as multicore CPUs, distributed-memory architectures, and graphical processing units (GPUs). The input data formats, preprocessing, and visualization are also presented.

In section VI, we outline the challenges in designing proteogenomic algorithms and the potential bottlenecks. In section VII, we present the existing HPC technologies in proteogenomics and how they are limited and do not scale properly. In section VIII, we conclude the paper and provide discussion and recommendations for future developments of big data high-performance tools in section VIII-A.

## II. PROTEOMICS AND PROTEOGENOMIC WORKFLOWS: CURRENT PRACTICES

**FIGURE 1.**
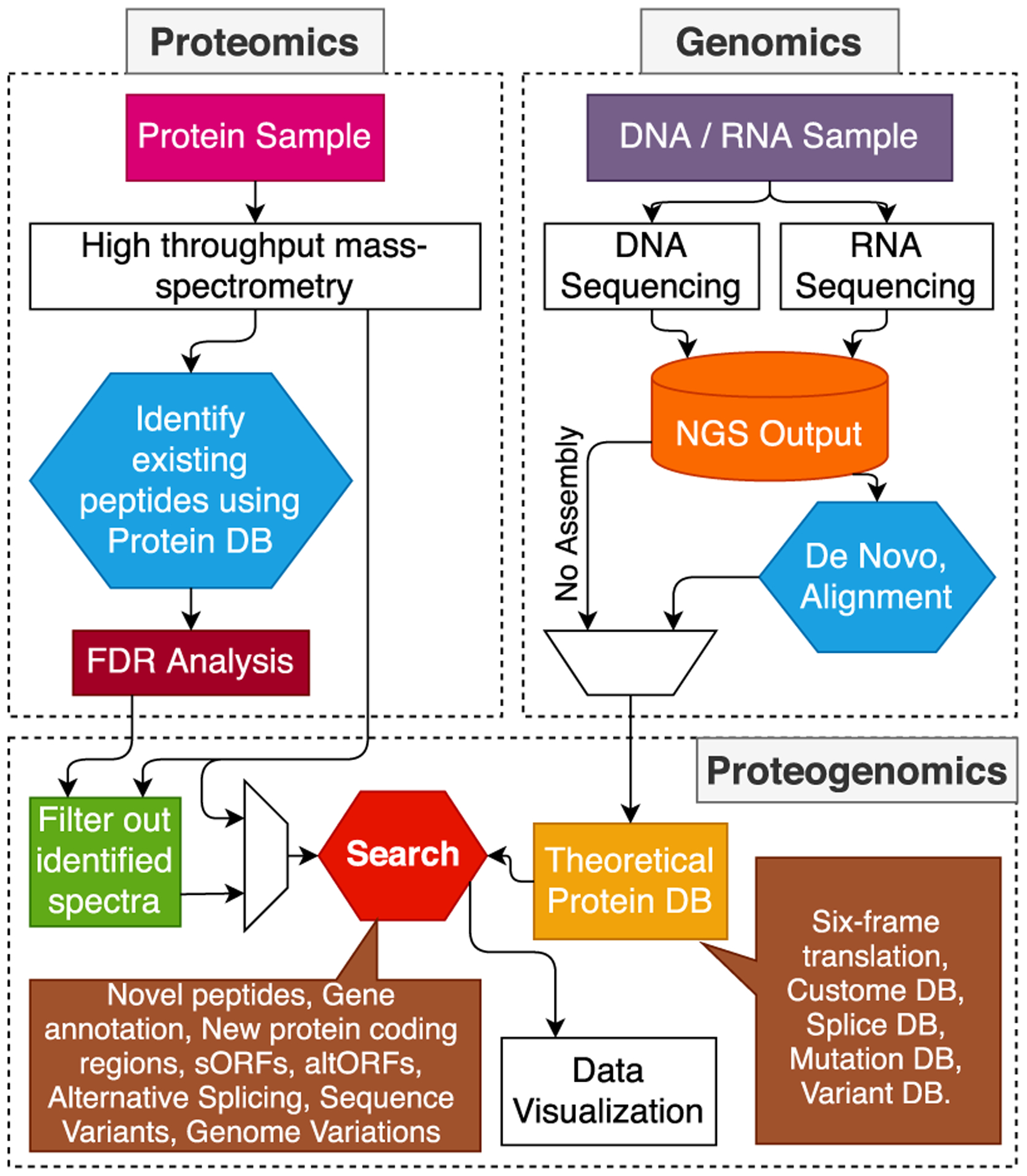
Proteogenomic Workflow. The proteomics workflow (left) shows different steps involved in peptide sequencing. A protein sample is processed and passed through a mass-spectrometer to get mass spectra of peptides. These spectra are then sequenced using either *de novo* sequencing or database search. Genomics workflow (right) shows the NGS sequencing of DNA (or RNA). The NGS reads can either be assembled into a genome and then converted to a theoretical amino-acid sequence database or six-frame translated without assembly to get better throughput of the entire proteogenomic pipeline. Once the amino-acid sequence database is ready, spectra are searched against this database to look for potential novel peptides.

In a systems biology setting, frequently used workflows (or pipelines) consist of a sequence of loosely connected computational tasks. *Database-search* workflows are the most commonly used data processing pipelines that require matching high-dimensional noisy MS data (called spectra) to a database of protein sequences. The entire workflow, scheduled using a script like structure, executes different algorithms, which run on a dedicated workstation. The data volume can easily reach the terabyte level depending on the experiment and search-parameters for these workflows. The currently used state-of-the-art serial and parallel methods are mostly data-oblivious, which can be detrimental for big data analytics. The proteogenomics workflow(s) comprises different stages, some of which are responsible for processing genomics and proteomics data. In contrast, others combine the data to report peptide inference and statistical confidence analysis of the identified peptides. Figure 1 shows a simple proteogenomics workflow consisting of standard processing stages.

As seen in Fig. 1 of the proteomics branch, experimental spectra are generated from a biological sample (a mixture of different proteins) by first digesting those proteins, usually by trypsin, breaking them into smaller peptides. After performing depletion and enrichment processes, the mixture passes through different mass-spectrometer stages, i.e., ionization, acceleration, deflection, and detection, to get MS/MS spectra. Analysis of this MS data is performed by searching the experimental MS spectra against theoretical spectra. These theoretical spectra are generated by digesting the custom protein database, which may have been generated from the genome sequences. For proteogenomics studies, NGS output can be used directly without assembly [45] or can be assembled into the genome using different assembling methods [46], [47].

Integrating the two (genomics and proteomics) branches shown in Fig. 1, different computational tools are used to search experimental spectra against a custom protein database [48]-[52]. By performing in-silico digestion of the database and generating theoretical spectra from the resulting peptides, these tools can compare the experimental spectra against the theoretical ones. The comparison is done either by peak-counting or performing a dot product between an experimental and theoretical spectrum to get a similarity score. The peptides identified in this way can either be intergenic, i.e., mapping to areas in the DNA located between annotated genes, or intragenic, mapping to areas in the DNA located within an annotated gene. For the later, the identified peptide can map to an exon, intron, exon-exon (alternative) junction, exon-intron junction, 5’ UTR or 3’ UTR sites [40]. Proteogenomic analysis of prokaryotes, e.g., bacteria is quite different from eukaryotes as splicing is rare in the former while they do use alternative start codons. This makes proteogenomics analysis even more suitable for prokaryotes.

The peptide database search approach works fine for modal organisms with correctly annotated reference proteome in an ideal setting. However, issues with peptide identification processes are well-known and prevalent, including miss-identifications of peptides, statistical inaccuracies of FDR analysis, and inconsistencies between different search engines [53]. Further, the vast majority of living organisms do not have their complete proteome available, and such databases with missing or incorrectly annotated peptides lead to incomplete results. There are many workarounds to this problem in the literature, e.g., *de novo* sequencing tries to construct the peptide sequence from the given spectra without any database search [54] or sequence tag extraction where small sequence tags are extracted from the spectrum and the search space is filtered using those tags [55]. Even with these filtering and search-space reduction techniques, our preliminary experiments suggest that most of the existing tools scale extremely poorly with the increasing size of the data or search space. Further, comparison across literature indicates decreased maximum accuracy of *de novo* algorithms (< 35%) [56]-[58] relative to database search algorithms (30–80% depending on the database and spectra) [59]. There is no single strategy that can claim to be the most accurate or the most scalable one.

## III. GENOMICS

The Human Genome Project (HGP) was completed for the very first time in the year 2003 (though the initial draft was published in 2001) [60] which involved the collaboration of researchers all around the world and took roughly 13 years. The overall cost of the project was about 2.7 billion dollars. This cost has been dramatically decreasing, surpassing Moore’s scale in 2008 when sequencing centers adopted Next Generation Sequencing (NGS) as opposed to traditional Sanger based sequencing.^1^ Here, we briefly discuss the four generations of sequencing technologies. Though genomics is a vast field, we discuss only the parts relevant to mass-spectrometry-based proteogenomics, i.e., 1) NGS Sequencing Techniques; 2) Genome Assembly, and 3) Customized database generation.

### A. NGS SEQUENCING TECHNIQUES

Each generation of sequencers came with its own set of advantages and challenges, e.g., it was faster and produced more genomic data, but the computational demands for analyzing these data sets also increased. Below we briefly outline the kind of sequencers that are currently available used for large-scale proteogenomics data generation.

#### 1) FIRST GENERATION

One of the classic examples of sequencing technologies is the Sanger based sequencing, which used chain termination methods and was developed in 1977 [61]. Though relatively slow, it is still used in a variety of applications, especially where high throughput is not required. On average, this methodology produces reads of length 600–1000 [62]. Another method, appropriated to the first generation of sequencing, is called Maxam-Gilbert [63], which was presented in 1980. Although it gained rapid popularity, its wide-spread use dropped subsequently due to numerous reasons, including extensive use of hazardous materials like Hydrazine, time-consuming and complicated processes, and the development of other simpler and faster techniques.

#### 2) SECOND GENERATION

Methods that belong to the second-generation sequencing mark the start of the Next-Generation Sequencing era. Sequencing by Synthesis or SBS, which is an improvement on Sanger based sequencing, is the most popular category of the second generation of sequencing methods. These methods improve over traditional techniques by massively parallelizing sequencing processes sequencing millions of fragments of 300–500 bases simultaneously. High error-rate is accommodated by using the majority consensus sequence as millions and billions of reads can be produced in one run [64].

Two popular SBS technologies are:

- **Ion Torrent** uses semi-conductor chips to directly translate DNA sequence into digital information [65]. It detects the changes in the solution’s pH levels due to the release of a hydrogen ion every time a nucleotide is added to the growing complementary DNA chain.
- **Illumina** sequencing uses bridge amplification to isothermally amplify each DNA fragment molecule to generate clonal clusters of about 1000 template molecules [66]. In the synthesis stage, nucleotides are modified with different fluorescent labels attached to the growing chain (complementary to the template), which emit a different fluorescent signal when excited by a light source. The base call is determined based on the intensity and the wavelength of the signal.^2^

#### 3) THIRD GENERATION

The third generation of sequencing marks the trend shift from the “short-reads,” popularized with the second generation sequencing, towards “long-reads” while maintaining the high-throughput of the process. The most prominent technology in this field is PaCBio’s Single Molecule Real-Time (SMRT) [67] used by the latest Sequel II system.^3^ HiFi reads (long reads with higher accuracy and throughput) of length 15–20 KBS or more with accuracy greater than 99% are generated with the total number of reads reaching as high as 4-Million. A higher rate of stochastic errors is usually fixed by sequencing the template multiple times and obtaining the consensus sequence.

#### 4) FOURTH GENERATION

Nanopore-based sequencing also referred to as the fourth generation of sequencing after its commercialization by Oxford Nanopore Technologies presents a radically different sequencing approach. In this technology, the entire DNA molecule is “ratcheted” through a protein nanopore embedded in an electrically resistant membrane [68]-[73] or a metal-alloy with pores of nanometer size in the case of solid-state technology [74]. Nucleotides in the sequence move through the nanopore, creating a disturbance in the ion-current flow, which is translated to the DNA sequence. The analysis can be performed at run time as the output can be streamed to a local computer as soon as the experiment starts. Using this technology, a handheld device (MinION^4^) can sequence the whole genome with reasonable accuracy.

Since the entire DNA molecule is sequenced, output reads’ lengths vary and depend on the sample preparation reaching up to 2Mbs with single-molecule accuracy of 95% and consensus accuracy of 99.999%. The experiments can quickly scale by running multiple flow-cells simultaneously (e.g., in PromethION) with throughput reaching as high as 10.5 Tbs in a single run.^5^

Today, a draft human assembly can be done under $100 [75] and the entire process takes about 6 hours to complete. The low cost of sequencing the entire genome is one of the primary reasons behind the popularity of sample-specific proteogenomics, where a customized protein database is generated for the sample under investigation. This involves whole genome sequencing, exome sequencing, and RNA sequencing. Using a sample-specific database also has the advantage of accounting for mutations, and SNPs specific to the sample, e.g., in oncoproteomics [76].

### B. GENOME ASSEMBLY

Once the reads are obtained from one of the sequencing methods discussed above, they are assembled in a *de novo* fashion to reconstruct the original sequence. For well-annotated species, a wild-type genome can be used in mapping the reads (and construct the genome) and identify sequence variants.

#### 1) DE NOVO ASSEMBLY

In *de novo* assembly, the short reads are aligned to form longer reads called contigs. The contigs can then be reordered and combined to form scaffolds to account for the gaps between contigs.^6^ Different assembly methods are employed based on the sequencing techniques used and the type of raw data available (e.g. short-reads or long-reads).

Common methods for assembling short-reads into longer contigs include::

- **De Bruijn Graph** constructs a graph by first splitting reads into smaller k-mers and then drawing an edge from the left k-1-mer to the right k-1-mer [77]. The edge corresponds to an overlap of size k-2. The sequence is determined by performing a modified Eulerian walk on the resultant graph. Two popular assemblers that use de Bruijn graph are Velvet [46], and ABySS [47].
- **Overlap Layout Consensus** constructs a graph by finding the overlapping of all pairs of reads. Since this requires multiple sequence alignments, the process only works for small datasets and does not scale well. Edena [78] assembler uses overlap layout consensus algorithm for genome assembly while SGA [79] use a similar approach called string graph [80].
- **Greedy and Hybrid Approaches** Greedy algorithms start by combining the best matching strings and use heuristics to approach the solution faster. On the other hand, hybrid approaches combine different algorithms to minimize the error. SSAKE [81] and PERGA [82] use greedy approach for assembly while Ray [83] uses the hybrid approach.

Tools for assembling modern-day long-reads include the most recent and fast wtdbg2 [84] which is 2–17 times faster than the next best algorithm assemble reads from both PacBio and Oxford Nanopore Technology sequencing. It uses methods like overlap-layout-consensus and improves the speed of all-to-all comparisons among reads and layout algorithms using the fuzzy-Bruijn graph. Other popular algorithms for long-reads include CANU [85] developed for PacBio’s SMRT, FALCON [86], Flye [87], and MECAT [88].

#### 2) MAPPING

The reads can be mapped to the reference genome to identify sequence variants. In case of low sequence depth and the availability of a closely related reference, genome mapping can also be used for assembly. However, the original sequence is not always known, especially for non-model organisms. The alignment can be done using different available tools e.g. Bowtie [89], BWA [90], Segemehl [91] etc. Minimap2 [92] is an efficient mapping tool for mapping long reads to reference genome.

### C. CUSTOMIZED DATABASE GENERATION

As different modifications and mutations that are sample-specific are not reflected in the reference protein databases, proteogenomics uses a custom protein database to search for different types of peptides, e.g., novel peptides, chimeric peptides, splice junction peptides, and intron peptides. However, for proteogenomics studies, this must be produced using genomics or transcriptomics data. Here we outline some of the ways a custom protein database can be generated.

#### 1) SIX-FRAME TRANSLATION

**FIGURE 2.**
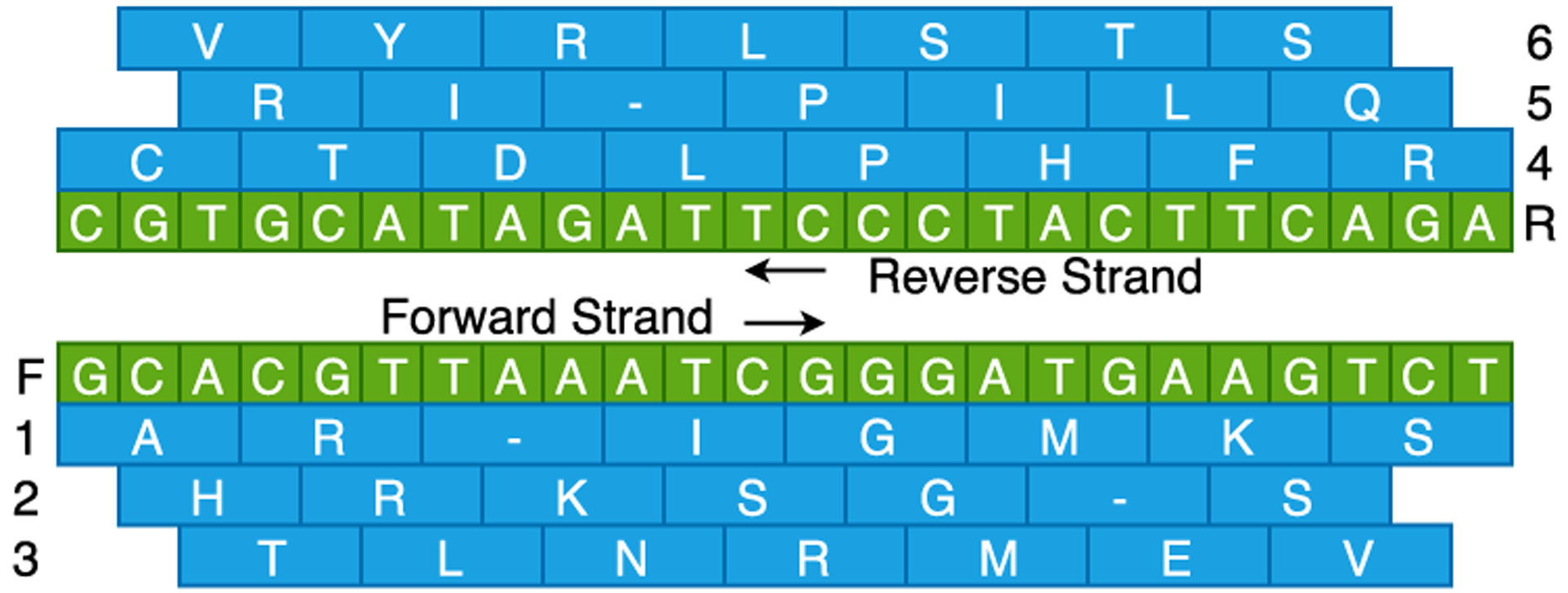
Six-frame translation of sample DNA sequence. The bottom half shows the forward strand and translation of its corresponding three frames. The top half shows the reverse strand and the translations of its three frames. Each green box represents a nucleic acid, and each blue box represents an amino acid. A blue box with ‘-’ shows that the corresponding codon is a stop codon.

Six-frame translation is probably the easiest method to generate protein sequences from DNA fragments. In this method, a theoretical database is created by translating all the six frames of the genome. Stop codons are translated to special characters (e.g. ‘-’, ‘*’), which are later used, during the search, to break down the amino acid sequences for further in-silico digestion. The translation is done using multiple codon charts (e.g., human and mitochondrial codon charts for humans) where a triplet of nucleic acid codes for one amino acid. One advantage of this approach is the thoroughness as nothing is left out. This can help identify novel peptides, especially in regions that are not expected to code for proteins. On the other hand, it creates a huge theoretical protein database with an excessive search-space footprint. It poses challenges in terms of computing resources and statistical confidence of the search results. A sample six-frame translation is shown in Figure 2.

#### 2) EXOME DB

Exome sequencing sequences part of DNA that codes for proteins. This part of the DNA can be converted into a protein sequence using six-frame translation. This process involves selectively capturing exons before sequencing and can significantly reduce the size of the theoretical database (since exome constitutes only a small amount of genome (less than 1%) in the case of humans) as well as help identify protein variants at significantly reduced cost [93].

#### 3) OTHER METHODS

The theoretical protein database can be reduced in size by filtering the genome using different methodologies that predict coding portions of DNA [94]. RNA-sequencing is also used to theoretically generate sample-specific protein database and search MS/MS spectra in the generated database [40]. Similarly, the database can also be created using *de novo* peptide sequencing or predicted from related species’ annotation.

## IV. PROTEOMICS

There are two approaches to analyze and characterize proteins in a complex sample using mass-spectrometry based techniques. The first technique is called the *Top-Down* approach, where the purified protein sample is directly fed to the mass-spectrometer where the proteins are ionized, fragmented, and detected. The result is a tandem mass spectrum, which is analyzed using computational methods to identify proteins and their modified variants in the sample. The protein may further be separated and fragmented (MS3) into peptides which may be analyzed to localize modifications to parts of the protein [95], disambiguate protein identification through sequence tags [95], improve on confidence score of identifications [96], help differentiate highly similar proteins [97], and perform similar targeted (MS^*n*^) analyses [98], [99].

**FIGURE 3.**
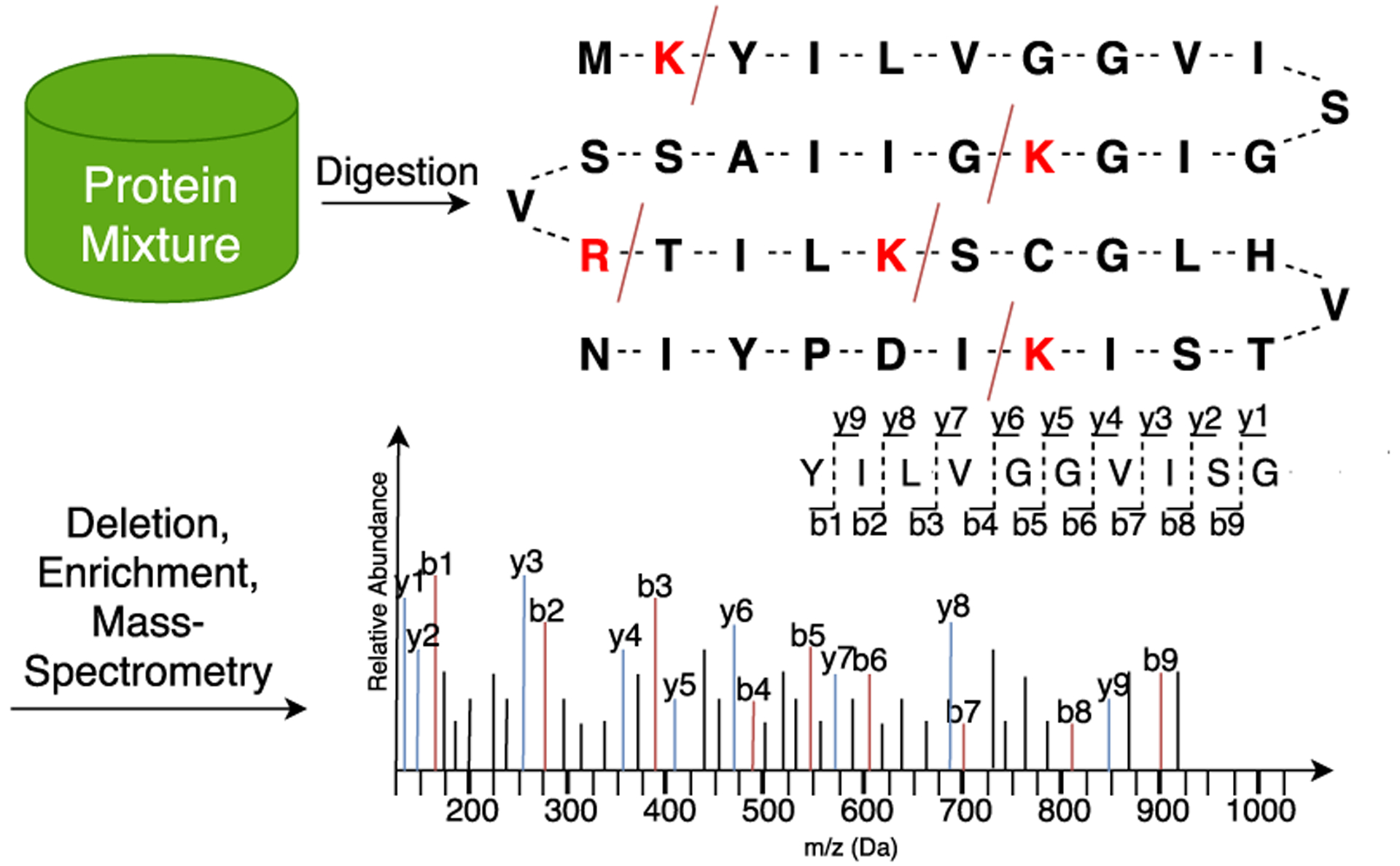
Process of generating MS/MS spectra from a protein mixture using mass-spectrometry analysis. Protein in the mixture is broken into peptides using the enzyme called trypsin, which breaks the protein strings at K and R bases generating peptides of varying sizes. This peptide mixture is then refined, and peptides are moved through a mass spectrometer, which generates an MS/MS spectrum for each peptide of a different mass.

The second and most commonly employed technique is called the *Bottom-Up* approach or Shotgun approach. In this case, the purified protein sample is first proteolyzed into a peptide mixture using an enzyme such as Trypsin. The peptide mixture is then fed into an automated liquid chromatography mass spectrometry LC-MS/MS pipeline for separation and fragmentation. The peptides in this pipeline are first ionized, and the first stage MS1 spectrum is acquired. Based on the data acquisition method, the peptide ions are selected and fragmented to obtain the mass-spectra of each or a group of co-eluting peptide ions. A simplified mass-spectrometry pipeline is shown in Figure 3.

Computational tools then process the resultant (MS/MS or MS2) spectra in an either untargeted or targeted manner to identify either all possible or specific peptides in the sample, respectively. The two data acquisition methods in the shotgun LC-MS/MS pipeline include (1) Data Dependent Acquisition (DDA) and Data Independent Acquisition (DIA).

### A. DATA DEPENDENT ACQUISITION (DDA)

In Data Dependent Acquisition (DDA), the peptide eluting from the liquid chromatography (LC) at a given point in time undergo mass-spectrometry where the peptide ions are detected in the first level mass spectrum (MS1). Then, only the *k* (~ 10 to 20) most abundant peaks (peptide precursor ions) are separated based on their m/z and selected for further fragmentation, hence data-dependent, in order to obtain second level MS/MS spectra. The advantage of DDA is the high-quality separation of peptide ions in the first stage, which yields unconvoluted MS/MS spectra where each fragment-ion is directly correlated to its peptide precursor ion. However, the disadvantage of this technique is the poor reproducibility since the intensities of eluted peptide ions in the first stage of mass-spectrometry may differ across experiments [100]. Further, since many peptide ions in MS1 are discarded, the obtained data does not entirely cover all the sample peptides/proteins. Finally, if a more significant number of peptide ions are chosen for fragmentation in MS1, then separation of each peptide ion and fragmentation may slow down the data acquisition speed.

### B. DATA INDEPENDENT ACQUISITION (DIA)

In Data Independent Acquisition (DIA), all peptide ions in the MS1 spectrum within a given window of m/z are chosen for MS2 fragmentation [101]. The method in which all ions entering the mass spectrometer at a point in time is chosen for fragmentation is called broadband DIA. A common method for broadband DIA is *MS*^*E*^ where a peptide is first subjected to low energy CID followed by high energy fragmentation [101]. Another variant of the DIA method is Sequential Window Acquisition of all Theoretical Mass Spectra (SWATH-MS) [102] which isolates windows (swaths) of a specific m/z range in the MS1 spectrum and fragments all peptides in each window for each time point in the chromatogram. The ion chromatograms for fragment-ions in MS2 spectra and their precursor ions are extracted, called extracted ion chromatograms (XICs), which allow a drastic improvement of signal-to-noise ratio in the multiplexed MS2 data. The advantage of SWATH-MS is that the swath windows can be fragmented in parallel, achieving high data throughput as well as enabling reproducibility and wide coverage of peptides in the sample [103]. Because of this, a complete digital archive of the original sample is formed for each time point in chromatogram as well as co-eluting MS2 spectra each window in each MS1 spectrum. The caveats of DIA based data is that the obtained MS2 data is highly convoluted since each MS2 spectrum now contains fragment-ions from multiple peptide precursors leading to loss of direct mapping between a fragment-ion and the precursor ion [101], [102]. A few spectrum-centric scoring-based techniques have been established to analyze SWATH-MS (DIA) data. These techniques require spectral libraries, which must be constructed from the same peptide sample using DDA methods [104]. The tools such as DIA-UMPIRE [100], DeMux [104], Group-DIA [105], MSPLIT-DIA [106] enable untargeted usage of substantially advanced DDA based peptide identification methods for DIA data by first detecting precursor and fragment-ion peaks from the multiplexed DIA spectra to construct pseudo-tandem DDA-like MS/MS spectra which are searched against a sequence database or spectral library for identification. PIQED [107] improves the previous methods to allow identification of post-translational modifications in the DIA MS2 data. DeepNovo-DIA [108] uses deep neural networks for *de novo* sequencing of peptides from DIA spectra data. On the other hand, Open-SWATH [109] allows a targeted analysis of the DIA data. An extensive survey on processing strategies and software solutions for DIA data is in [110].

### C. MS/MS DATA PREPROCESSING

The DDA MS/MS data obtained from the high-resolution mass-spectrometer instruments usually contain significant noise in spectra, which may be misconstrued as fragment-ions [111]. Further, it is also possible that the same peptide ion undergoes fragmentation multiple times, generating (similar) redundant MS/MS spectra for the same peptide. Therefore, it is pertinent to remove noisy peaks from spectra [112], remove low-quality spectra [113], [114], reconstruct the missing peaks in spectra [115], [116] and merge (cluster) redundant MS/MS spectra [117] before analyzing the data for peptide identification. It has also been shown that data reduction speeds up the peptide identification rate [114]. pParse [118] uses a machine learning-based method to extract spectra from co-eluting peptide precursor spectra and reconstruct missing complementary peaks in the separated MS/MS spectra. MS-REDUCE [112] uses random sampling, an approximate quantization-based technique to reduce the noisy peaks from the MS/MS spectra data. The spectral clustering tools such as MS-Clustering [117], Pep-Miner [119], CAMS-RS [120], MaRaCluster [121], and others [122] have been proposed for removing low quality and redundant MS/MS spectra from the dataset. A comprehensive survey of MS/MS data preprocessing techniques is also done in [111].

### D. PEPTIDE IDENTIFICATION

Several computational techniques for identifying peptides from the acquired (DDA) tandem MS/MS spectra data have been employed across the existing search tools. The computational techniques can be broadly classified into three main categories. i.e. *de novo*, spectral library search and sequence database search [123].

#### 1) DE NOVO

*De novo* peptide deduction methods are usually employed when there is no a priori reference spectral library available [124]. The peaks in the experimental spectra are labelled using amino acid masses, charge and fragmentation type information. The disadvantage with this method is the low identification rate since it is highly challenging to cater for post-translational modifications (PTMs), imperfect fragmentation and other mutations in peptide identification process. The existing *de novo* tools are PepNovo [124], pNovo [125], Open-pNovo [126], DeepNovo [56], PEAKS [127], Lutefisk [128], Spectral Networks [129], AuDeNs [130], MSNovo [131], SeqMS [132], PFIA [133], and NovoHMM [134]. The algorithmic workflow used in many *de novo* tools involves constructing graphs or networks where the nodes are the peaks selected from a spectrum while the acyclic optimal paths between the nodes are constructed using computational techniques such as dynamic programming based on probabilistic peptide fragmentation models. Though *de novo* method can be used in proteogenomics to identify novel peptides directly from the spectra, the limited accuracy has mostly restricted their widespread use. Recently, *de novo* sequencing along with advanced proteogenomics techniques was used to identify novel proteins/peptides, improving the existing reference database and gene annotations in *Sordaria macrospora* [135].

#### 2) SPECTRAL LIBRARY SEARCH

In case when a library of previously annotated MS/MS spectra are available, the acquired tandem experimental MS/MS spectra are compared against the spectral libraries using signal processing methods. The advantage of spectral library search is the high peptide identification rate as well as confident quantification of post-translational modifications [136]. However, the disadvantage is the availability and small size of spectral libraries. Further, the proteomics experiment must be performed under same conditions as the spectral library data. The existing spectral library search tools include ANN-SoLo [137], pMatch [138], SpectraST [136], Pepitome [139], NIST MSPepSearch [140], X! Hunter [141], ProMEX [142], HMMatch [143], MSDash [144], QuickMod [145] and MzMod [146]. These tools employs a scoring mechanism to compute the similarity measure between the reference and experimental spectra.

#### 3) DATABASE SEARCH

Finally, the most commonly employed method for peptide identification is the database search where the experimental tandem MS/MS spectra are compared to the theoretically predicted spectral libraries [52]. The theoretical spectral libraries are generated by first in-silico digesting a proteome sequence database into peptide sequences and then predicting MS/MS spectra for each peptide sequence and its possible (modified) variants. The advantage of this technique is that the PTMs and fragmentation types can be easily incorporated in the theoretical spectra. Furthermore, recently, a few machine learning based techniques [147]-[150] have been proposed that can predict intensities along with m/z’s of fragment-ions in the theoretical spectra which can substantially improve the peptide identification rate for sequence database search. A small list of peptide database search tools includes SEQUEST [49], MSGF+ [151], SpecOMS [152], MSFragger [52], Open-pFind [153], Andromeda [154], X! Tandem [155], Crux [156], Tide [157], Comet [51], TagGraph [158], PEAKS-DB [159], JUMP [160].

**FIGURE 4.**
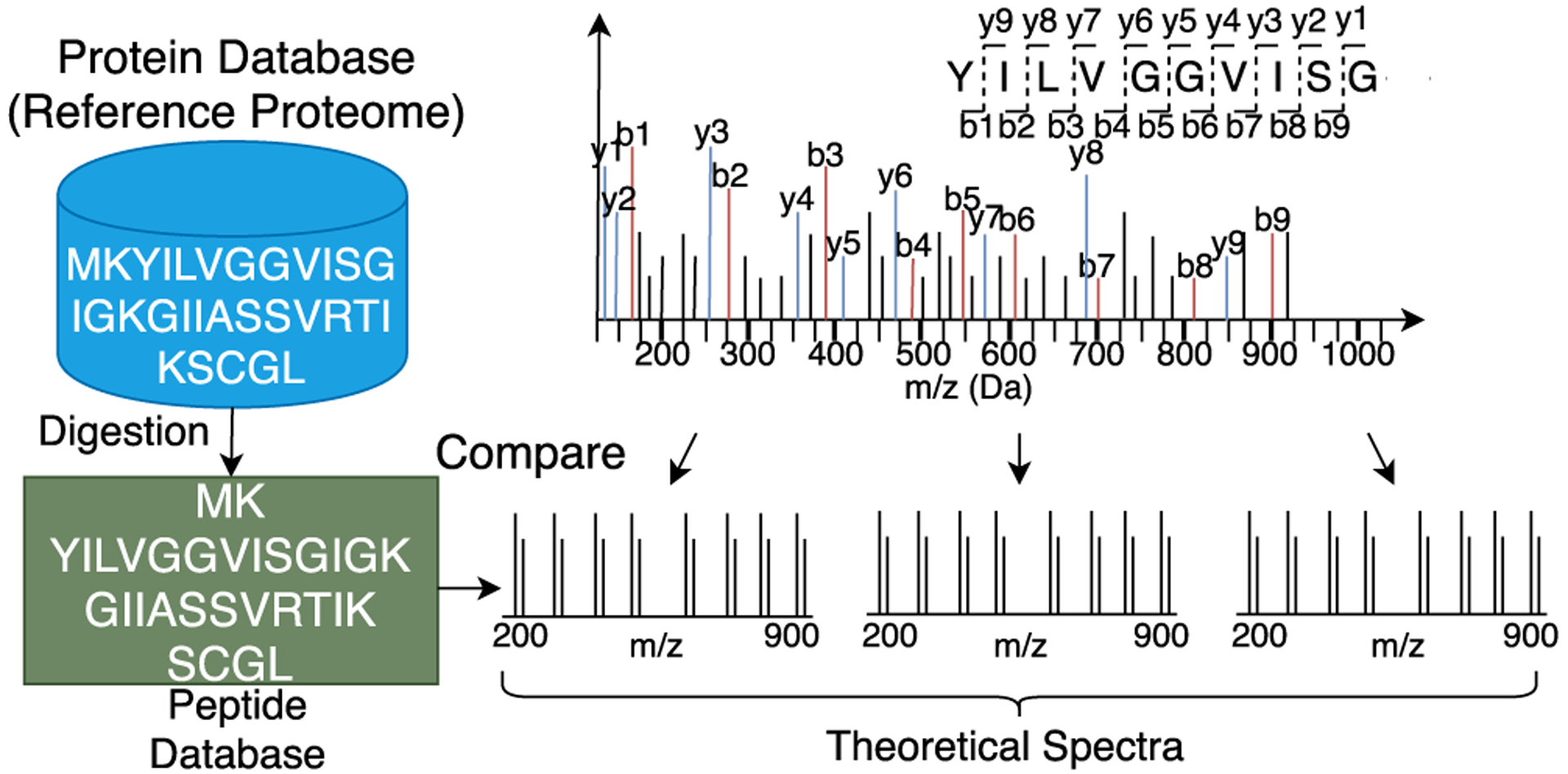
A generic proteomics flow. In-silico digestion of the protein database is performed to generate peptides. These peptides are then converted to the theoretical spectra and compared against the experimental spectra.

The protein sequence database often employs a combination of techniques for filtration of search space followed by peptide-to-spectrum match (PSM) computations. Further, since all-to-all comparisons in the spectral library and sequence database search are not feasible, a faster similarity metric such as shared-peak count [52], [152], [161]-[164], sequence tagging [55], [153], [158], [159], [165]-[169] combined with peptide precursor mass [51], [170]-[172] based methods are combined with precursor charge, enzyme specificity and other features in MS/MS data to filter the search space to only the relevant database or spectral library entries. The filtered search space is then formally scored for peptide-to-spectrum scoring is computed using a similarity score computation algorithm such as [49]-[51], [151], [155]-[157], [164], [173], [174]. A generic workflow of a protein database search is shown in Figure 4.

#### 4) POST-PROCESSING AND FDR ANALYSIS

Finally, the obtained peptide to spectrum match, spectrum to spectrum match, and the *de novo* constructed tag scores are re-analyzed to assign confidence to the computed scores. The confidence scores are assigned using probabilistic classification models that compute the probability of a match to be the result of random chance, called FDR, and discard low confidence matches. The conventional methods such as Percolator [175]-[177] and Peptide/Protein-Prophet [178], and iProphet [179] employ target-decoy based methods where the search is also conducted against a database of decoy proteins. Then a classification model is trained to separate the targets and decoys while assigning the confidence scores. Percolator uses a semi-supervised learning approach to train an SVM on a subset of high confidence PSM using several features, including match score, spectrum and peptide mass, mass difference, charge, and many others. On the other hand, PeptideProphet learns the distribution of search scores across the dataset to determine the correct matches. Other PSM/SSM ranking algorithms such as the ones explained in Supplementary Text of TagGraph [158] and Open-pFind [153] employ a decoy free model and use probabilistic distribution-based models using several features in the MS/MS data.

## V. PROTEOGENOMICS

The goal of proteogenomics is to perform an exhaustive search to discover novel peptides by searching each conceivable peptide, e.g., six-frame, splice graph, or other customized databases. One of the common ways to search for intragenic peptides is to perform a search by creating a theoretical protein database by direct six-frame translation of the genome. The memory requirements for these different steps are listed in Table 1.

This has certain limitations in terms of enormous database size, which mostly contains *non-existing* proteins leading to an inflated number of peptide matches and high false-positive rates, and the inability to identify exon-exon junction peptides. Some techniques have been designed to increase the accuracy of these algorithms for matching, such as creating a customized database for proteogenomic analysis and using filtration techniques to predict coding genes in the translated database. RNA-sequencing data can also be used to generate a more compact, customized protein database.

An ever-increasing number of tools for proteogenomics is being published as the interest in research grows. These tools range from performing just the essential tasks of analysis, i.e., searching spectra against a custom or six-frame translated database to complex multi-purpose pipelines that perform tasks like merging results from different tools, visualization, and automated annotation [4], [8], [13], [39], [180]. The first step includes obtaining a database against which the experimental spectra can be searched. This database can be constructed in a customized way, e.g., combining different annotated smaller database like pseudogene DB, lncRNA DB, splice DB, UTR DB, and Small Open Reading Frame (sORF) DB, or the entire genome can be translated to create a complete database with every possible amino acid sequence that could be generated. The next step is to search the experimental spectra against one or multiple databases to find novel proteins or peptides. To shortlisted candidate peptides, a search against the reference proteome is performed to filter out known peptides. This step can reduce the number of spectra to be searched in the more extensive theoretical database. Once the identified peptides are selected, they are further curated and screened to remove any false matches that might have passed the FDR filter [181]. As the search space for proteogenomics is enormous (hundreds of times larger than the reference proteome), the number of false PSMs that pass the FDR filter tends to be much higher than the specified threshold. Therefore, specific guidelines need to be followed when reporting proteogenomic matches as novel peptides [40].

Despite all the efforts, the inherent problems of proteogenomics are still dominant and a significant factor in limiting the progress in the field. These problems are three folds, i.e. **1)** The huge amount of data that needs to be processed and analyzed, e.g., the six-frame translated database for the human genome is 600-times larger than the reference proteome, which is further expanded with the choice of the search parameters, **2)** the amount of time it takes to process the data and search the mass spectra against the theoretical database, and **3)** the poor quality of the matches due to inflated search space which leads to a significantly large number of false-positives. Our experiments show that searching 1-million spectra against the complete translated human database using Peppy [8] takes about 15 days on a 24-core machine while only 50% of the reported PSMs (reported to be under 1% FDR) are actual matches. Most of the published tools are only tested on small datasets that are highly specific to their research groups, and no qualitative assessment is performed on popular benchmark datasets [182]. Also, there are no known quantitative frameworks that can be used for scalability or quality assessments.

### A. PROTEOGENOMIC TOOLS

A vast array of proteogenomic tools perform distinct steps in different stages of the proteogenomic pipeline. Some provide a complete analysis pipeline while others only focus on a certain part of it. For this study we only pick tools that provide end-to-end proteogenomic pipeline. Based on peptide identification, the proteogenomic tools can be divided into two broad categories i.e. *de novo* and database search. There are only a few tools that perform *de novo* deduction such as Peptimapper [183], IggyPep [184], and Pepline [185]. There is a wide variety of tools using different runtime environments, inputs, peptide search engines, scoring methods, FDR analysis, and visualizations in database search. Tools such as PGTools [180], Galaxy-P [39], ProteoAnnotator [186], IPAW [13], JUMPg [187], Graph2Pro/Var2Pep [188], NextSearch [189], and PGP [190] support execution on distributed memory environment using job scheduling frameworks such as PBS or Torque etc. Peppy [8], PGMiner [191], and PGA [192] only support multi-core processing while GenoSuite [193], Enosi [4], Bacterial Proteogenomic Pipeline [194], and MSProGene [195] only use single execution core. For the selection of our tools we consider the following components to be included in the pipeline:

- Generation of Theoretical Protein Database.
- Database Search and Peptide-Spectrum-Match (PSM) Scoring.
- FDR Analysis.
- Mapping Discovered Peptides to Genome.

Details of state-of-the-art end-to-end proteogenomic pipelines are given in Table 2 where we outline the availability of some of the features. Table 3 categorized these tools based on their type, run-time environment, and proteomic search engine used.

All of the workflows shown in these tables are not cache-aware. They do not consider the communication costs associated with the data movement within modern multicore memory-hierarchies, manycore architectures. The way memory-accesses are done can have a significant performance degradation for single machines and multiple distributed-memory machines. We do not know of any cache-aware (or memory hierarchy aware) algorithms for MS-based proteomics or proteogenomics. Therefore, parallel algorithms that can exploit spatial and temporal locality in processing MS-based workflows will be of utmost importance.

At a finer granularity level, the MS proteomics data involves irregular computations in which the objective of the analysis is to clean, analyze, and search the MS data. For example, when cleaning the MS data, the spectra are often normalized, clustered together, and partially reconstructed using a graph or machine-learning computations. Similarly, when searching the MS data against a database, the experimental spectra may share many candidate database peptides, especially in open-search. Moreover, the candidate database peptides for an experimental spectrum may not be placed in adjacent memory locations depending on the indexing and search methods. For example, a sequence-tag based search strategy may first sort the database entries by the shared kmer counts and then by precursor mass. Therefore, solving these computational problems requires data structure(s) such as hash-table, graphs, and sparse unstructured matrix computations that exhibit little memory-locality and naturally lead to unpredictable communication patterns both in space (arbitrary connections between computing components) and time (the processes or threads may need data from one another at any point in time). Further, the existing high-performance computing methods [196]-[198] have been designed over inherently serial designs where the database is replicated on all parallel nodes, and the experimental data are split among them. This strategy is not scalable due to the space complexity associated with indexing proteome databases with multiple PTMs (specially fragment-ion index) [199], or multiple proteome database searches are required for systems biology experiments.

In Table 4, we describe different visualization methods used by the tools.

## VI. COMPUTATIONAL CHALLENGES IN PROCESSING BIG PROTEOMICS AND PROTEOGENOMICS DATASETS

**FIGURE 5.**
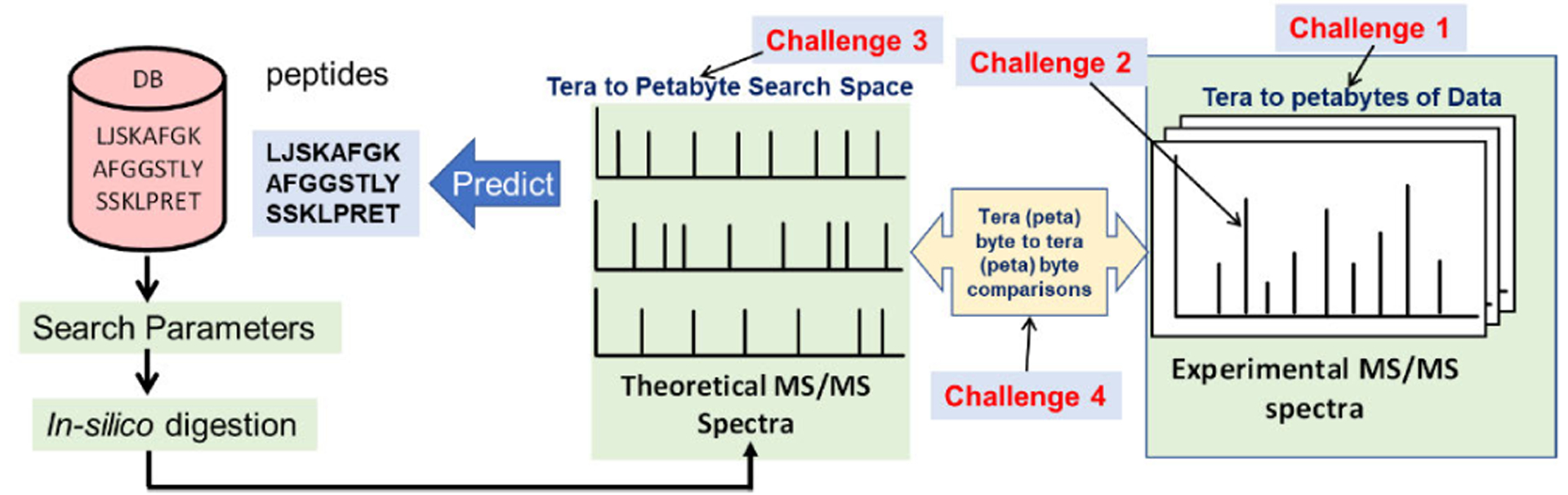
Scalability bottlenecks of current workflows for Mass Spectrometry based omics data analysis are identified. These bottlenecks are a major limitation for any MS based omics data anlaysis that has large number of spectra or big database (e.g. for proteogenomics).

Our experiments and thorough review of the literature give us insight into why most workflows have low scalability. All proteomics workflows try to get the MS data, which is then “matched” with the theoretical spectra obtained from the in-silico digestion of protein databases. A simplistic workflow is illustrated in Figure 5, which shows the reader the computational and the associated scalability challenges of the problem. Bottleneck 1 and 2 are associated with the big data sets produced by mass spectrometry instruments. Bottleneck 3 and 4 are associated with the way that the current workflows work and process the data.

### A. BOTTLENECK 1

Millions to billions of spectra for extensive scale systems biology studies [147] are generated. The volume of these spectra can easily reach the terabyte level, which is problematic for existing algorithms that are not designed for scalability. The newly minted timsTOF is expected to produce 3GB/hour of data [200].

### B. BOTTLENECK 2

The experimental mass-spectrometry data are often noisy and require pre-processing before they could be used for peptide identification. Several algorithms have been proposed for spectral pre-processing that employ *compute intensive* techniques such as numerical analysis, signal detection, intensity normalization, multiply charged ion detection, clustering, periodic background noise removal, and quality assessment [112], [114], [201], [202] to yield quality data. Further, with the recent advent in the machine- and deep-learning-based pre-processing algorithms [149], [203], the resource demands for this step are rapidly increasing. Repeatedly (millions of times) executing these pre-processing tasks on a hundred (to thousands) of peaks per spectra can be a severe bottleneck.

### C. BOTTLENECK 3

The space complexity of the theoretical database (used for matching) is dependent on the search parameters and is significantly inflated as more and more post-translational modifications are incorporated. The standard human proteome database (30MB) can produce a 1TB theoretical search space (considering fragment-ion indexing) if only the ten most common PTMs such as oxidation, phosphorylation, amidation, acetylation, etc. are included. The complexity gets even worse if the semi-trypict and non-specific peptides are also considered. Therefore, when a large experimental dataset is open-searched against these massive search-spaces, 10^12^ to 10^15^ level computations may need to be performed, even with modern methods such as shared peak counting, sequence tagging, and peptide precursor mass tolerance to correctly identify the dark-matter [204] of proteomics. This may result in unreasonably long processing times [199]. We have recently formulated a theoretical framework and showed that communication costs (between cache and processor, or between different processors on memory-distributed machines) are the biggest bottleneck in achieving a scalable framework for MS-based omics [205]. Eliminating this bottleneck will require next-generation communication-avoiding algorithms [205], [206] for MS-based omics data analysis.

### D. BOTTLENECK 4: (CURRENT SERIAL ALGORITHMS)

The current serial algorithms for MS-based omics [51], [52] [155], when developed, considered *arithmetic operations* as the sole metric for efficiency [205]. Over time, especially in the last decade, the technological trend of Moore’s law has kept making the arithmetic operations faster. Therefore, the bottleneck for many MS-based omics algorithms has shifted from computational arithmetic operations efficiency to *communication* i.e., communication costs of moving the data between different memory-distributed processors connected via a network. However, we are not aware of any algorithm that eliminates this bottleneck for the processing of MS-based omics data sets.

## VII. CURRENT HPC METHODS FOR ANALYSIS AND THEIR LIMITATIONS

Empirical scalability results that we have observed for a vast majority of proteomics and proteogenomics tools can be explained using the bottlenecks. One approach that might help alleviate some of the scalability issues is by using high-performance computing techniques. These techniques may include manycore, multicore architectures, CPU-Accelerator models, and memory-distributed clusters. However, the applicability of these techniques in regular workflows has been limited. A limited number of parallel computing solutions have been proposed. These parallel solutions use two abstractions for MS-based omics data analysis. In the following, we discuss the limitation of these abstractions.

### A. LIMITATION 1

Proteomics pipelines are built over inherently shared-memory methods. Some studies have shown speedups for the computation part of the algorithms (i.e., scoring of the spectra). The parallel design employed in most of these studies involves spawning multiple instances of the original shared memory code and splitting the experimental spectra among them. These studies include Parallel Tandem [196] which spawns multiple instances of the original X!Tandem on distributed machines; X!!Tandem [207] achieves parallelism using owner-compute MPI processes; MR-Tandem [208] uses Map-Reduce instead of MPI for better speedup efficiency; MCtandem [209] employs Intel Many Integrated Core (MIC) architecture co-processor to speedup spectral dot products (SDP) for X!Tandem, and SW-Tandem [197] employs the Haswell AVX2 engine to speedup SDP computations on Sunway Taihulight supercomputer. SW-Tandem also spawns a manager process that distributes the experimental data to worker processes using a global queue for better load balancing. Bolt [198] constructs the complete fragment-ion index at all worker nodes and splits the experimental dataset among them. Therefore, the parallelism achieved by these algorithms is limited (30x speedup for 2000 cores [208]) and has not found widespread usage. Communication is a significant bottleneck for MS-based omics data analysis. The design and development of next-generation communication-avoiding HPC algorithms will be needed to alleviate these limitations [205].

### B. LIMITATION 2

Only a small number of proteomics and proteogenomics pipelines (both serial or HPC) take advantage of the heterogeneous multi-layered parallelism available on modern architectures in an end-to-end fashion. This includes many cores architectures (such as Intel Phi), CPU-Accelerator architectures (such as CPU-GPU [210] and CPU-FPGA [211]), vectorization in multicore systems (exploiting SIMD/MIMD), and Memory-level parallelism/instruction-level parallelism using software/hardware co-design. A carefully designed parallel algorithm for big data must include provisions for memory bandwidth utilization, communication costs, and cache awareness, which are missing from the current implementations [205]. This leads to limited speedups with the increasing number of processors or data and sub-optimal utilization of massive ubiquitous parallelism available on modern architectures.

## VIII. CONCLUSION

To date, the processing of high-throughput Mass Spectrometry (MS) data is accomplished using serial algorithms. Developing new methods to process MS data is an active area of research [52] but there is no *single* strategy that focuses on **scalability** of MS-based methods [212]. Mass spectrometry is a diverse and versatile technology for high-throughput functional characterization of proteins, small molecules, and metabolites in complex biological mixtures. In recent years the technology has rapidly evolved and is now capable of generating increasingly large (multiple terabytes per experiment) [52] and complex (multiple species/microbiome/high-dimensional) data sets [213]. This rapid advances in MS instrumentation must be matched by the equally fast and rapid evolution of scalable methods developed to analyze these complex data sets. Ideally, the new methods should leverage the rich, heterogeneous computational resources available in a ubiquitous fashion in the form of multicore, manycore, and CPU-GPU architectures. The absence of these high-performance computing algorithms now hinders scientific advancements for mass spectrometry research [212].

This study analyzes complete standalone proteogenomic pipelines in terms of their performance with increasing input size and varying input characteristics. Proteogenomics is an emerging research field and getting increased attention in the bioinformatics community due to the perpetually shrinking cost of NGS sequencing and high-throughput tandem mass spectrometry. Integration of these two techniques and the availability of high-performance computing can lead to the realization of performing a comprehensive analysis to find novel peptides. Our experiments have shown that the state-of-the-art tools exhibit poor scalability and low-quality results due to large search space when using genome translated databases. In this article, quantitative experimentation and analysis are provided for the performance of these tools in terms of time and memory. Quality degradation analysis, in terms of increasing database size, is also provided. With in-depth analysis and controlled experiments, we show that these tools are not sufficiently scalable for big data sets, and more expertise in programming and high-performance computing is required to build proteogenomic tools that can scale with the increasing size of data and can exploit heterogeneous HPC architectures for scalable executions.

### A. DISCUSSION AND RECOMMENDATIONS

The existing state of the art tools for analyzing proteogenomics data have largely not considered the scalability of the algorithms with the increasing size of the data. No single algorithm can be considered the most accurate or most scalable, even with a plethora of proteogenomics data analysis tools that have been proposed, designed, and developed. Our experiments have shown that Peppy, a state-of-the-art tool, takes more than 16 days to complete computations for 1-million spectra when memory is limited to 48 GBs. For reference, we executed Peppy on a server with more than 500 GB of memory and similar computation power to the previous one to determine the performance offset due to memory constraints. The runtime for the new server was nearly halved (8 days compared to ≥ 15 days) with maximum memory consumption of 150 GBs. These preliminary results further show that even when the tool depicts the linear increase in memory for small datasets, this assumption does not hold when the size of the data is increased. This is likely due to the poor decomposition of the database and spectra when the entire dataset does not fit into memory. Peppy slices the database into smaller chunks and searches the entire spectra set against each of these chunks. This requires multiple writes of the same chunk of data to the memory, which adds the additional overhead. Furthermore, searching the entire spectra set against the whole database is highly inefficient for multiple reasons, i.e., the amount of time it would take to search the data and the waste of bandwidth due to multiple writes to memory and the increased probability of high scoring random matches. The same kind of behavior can be witnessed for tools such as Tide or MSFragger.

Considering the oversights and shortcomings in proteogenomic and proteomics tool development for big data, we present some recommendations that we consider imperative for future designs and ensure that the proteomics methods remain scalable.

#### 1) EFFICIENT MEMORY-HIERARCHY AWARE DESIGNS

All of the current algorithms that deal with proteomics and proteogenomics analysis assume that all data is going to fit in memory. However, modern memory-hierarchies are complex and require a careful design for scalable implementations. The current computing machines are complex-systems that interchange the data between various levels of cache (with different sizes and speeds), translation look-aside buffers (TLB), persistent memories, virtual-memories, and hard-drives. The delays caused due to instruction-cache misses, data-cache misses, or TLB misses can significantly deteriorate the implemented code’s performance, even assuming that the data will fit in memory. Therefore, proteomics and proteogenomic algorithms must be designed to exploit the spatial and temporal regularities in accessing and computing the data.

#### 2) HIGH-PERFORMANCE COMPUTING STRATEGIES

Most of the software implementation of proteogenomic and proteomics tools has been with the assumption of a powerful (single-core) CPU. However, with Moore’s law limits, almost all desktop systems available possess some multicore architecture. Any performance improvement depends very much on the software algorithms and their implementations. In particular, gains are limited by the fraction of algorithms that can run in parallel on multiple of these cores. Although one might think it would be straightforward to parallelize MS-based proteomics algorithms since MS spectra themselves are small and could be blazed through the two (or four) cores to exploit embarrassingly parallel strategy leading to maximization of performance benefits. However, database search pipelines are interdependent linking of workflows that require data from non-contiguous memory spaces. This results in squandering of parallelization that may be available on multicore systems for these proteogenomics workflows, leading to sub-optimal speedups and performances.

#### 3) EXPLOITING HPC STRATEGIES FOR HETEROGENEOUS ARCHITECTURES

The level of heterogeneity in our desktops and laptops are gradually increasing. This is due to more number of cores (may be different or same), including Graphical Processing Units (GPUs), Field Programmable Gate Arrays (FPGA), System-on-Chips (SoC). Such heterogeneity in the processing units can lead to an enormous increase in efficiency but may present new challenges not found in typical homogeneous systems. Maximizing exploitation of these parallel systems necessitates optimal partitioning, minimizing surface-to-volume ratios, catering for the non-uniform processing power of different components as well as non-uniformity in the computational load to be incorporated in the design of these proteomics pipelines. Efficient design and implementation of these workflows will ensure scalability of these proteomics pipelines with increasing capability of Mass Spectrometers and with increasingly complex analysis of proteogenomics and metaproteomics analysis.

Other needs such as ensuring big-data management pipelines, generalized, open-source, managed platforms, and formatting rules for MS data reading and writing would ensure that the developed tools have a long-lasting impact on scientific investigations.

## Figures and Tables

**TABLE 1.** Various stages of Spectra-to-Peptide Database Search Work-flow. Note that the indexed size of the database depends on the initial size of the proteome database and the post-translational modifications added that could inflate the size to terabytes. Typical output file sizes for other steps such as pre-processing, scoring, FDR, quantification are used.

Step	Output Format	Output size/Memory Requirements	Software
Mass Spectra Generation	Thermo Fisher RAW files	several GB (LTQ Qrbitrap); timsTOF (3GB/h) - TB	Mass-Spec instrument’s software
Pre-processing of Spectra	mzML mzXML MS2	several GB, TB for timsTOF	MS-Reduce, Tide MS-GF+ MSFragger pFind
Protein Digestion	FASTA	few MB - GB	Protein Digestion Simulator OpenMS
Sorting/Indexing of database	N/A	several GB - few TB	MSFragger pFind-Alioth
Scoring & Peptide deduction	PEPXML	few MB - GB	Comet X!Tandem MSFragger MS-Amanda
False Discovery Rate	POUT XML PEPXML	few MB - GB	Percolator Peptide Prophet
Quantification of Peptides	XML	few MB - GB	Various

**Table 2.** Complete proteogenomic pipelines and support for certain features. Genomics: The tools to process different types of input data, i.e., RAW NGS, Aligned NGS, Reference Genome, and/or Transcriptome, along with the generation of different types of databases, are provided. Proteomics: The ability to filter out spectra of known peptides by searching first in the reference proteome is highlighted. The availability of the pre-processing feature, clustering, type of scoring, and FDR is also provided.

Tool					Genomics			
	Maintained	HPC	RAW NGS	Aligned NGS	Genome	Transcriptome	6FT	Customized-DB
Peppy	✗	✗	✗	✗	✓	✗	✓	✗
PGTools	✗	✓	✗	✗	✓	✓	✓	✗
Galaxy-P	✓	✓	✓	✗	✓	✓	✗	✓
IPAW	✓	✓	✗	✗	✓	✓	✓	✓
ProteoAnnotator	✗	✓	✗	✗	✓	✗	✗	✗
GenoSuite	✗	✗	✗	✗	✓	✗	✓	✗
Enosi	✗	✗	✗	✗	✓	✗	✓	✗
Bacterial Protoegenomic Pipeline	✗	✗	✗	✗	✓		✓	✗
JUMPg	✓	✓	✓	✓	✓	✓	✓	✓
PGMiner	✓	✓	✗	✗	✓	✗	✓	✗
NextSearch	✓	✓	✗	✗	✓	✓	✗	✓
Graph2Pro Var2Pep	✓	✓	✓	✗	✓	✓	✗	✗
PGP	✗	✓	✗	✗	✓	✗	✓	✗
MSProGene	✗	✗	✗	✗	✓	✓	✓	✓
PGA	✗	✓	✓	✗	✓	✓	✓	✓
Tool					Proteomics			
	ML	Visualization	Proteome	Preprocessing	Clustering	Scoring	FDR	Mapping
Peppy	✗	✗	✓	✓	✗	Peppy	TD	✓
PGTools	✗	✓	✓	✗	✗	xcorr	TD	✓
Galaxy-P	✗	✓	✓	✗	✗	Paragon	TD	✓
IPAW	✗	✓	✓	✓	✗	dot-product	TD	✓
ProteoAnnotator	✗	✓	✓	✗	✗	hyperscore	TD	✓
GenoSuite	✗	✓	✓	✗	✗	hyperscore	TD	✓
Enosi	✗	✗	✓	✗	✗	MSGF+	TD	✓
Bacterial Protoegenomic Pipeline	✗	✓	✓	✗	✗	NA	NA	✗
JUMPg	✗	✓	✓	✓	✗	JUMP	TD	✓
PGMiner	✗	✓	✓	✓	✗	hyperscore	TD	✓
NextSearch	✗	✓	✓	✗	✗	MSGF+	TD	✓
Graph2Pro Var2Pep	✗	✗	✗	✗	✗	MSGF+	TD	✓
PGP	✗	✓	✓	✓	✗	MSGF	TD	✓
MSProGene	✗	✓	✓	✗	✗	MSGF+	TD	✓
PGA	✗	✓	✓	✗	✗	Hyperscore xcorr	TD	✓

**TABLE 3.** Complete proteogenomic pipelines and their details.

Type	Environment	Input Data	Engine	URL	Tool
*De novo*	Single-core	MS/MS spectra (.mgf) Genome Database (.fasta)	PepNovo+	https://proteogenomics.page.link/peptimapper	Peptimapper [183]
		MS/MS spectra Genome Database (.fasta)	PepNovo v3 Mascot	Not available	IggyPep [184]
		MS/MS spectra (.mgf) Genome Database (.fasta)	Taggor (Built-in) Mascot	Not available	Pepline [185]
Database Search	Cluster	MS/MS spectra (.mgf) Genome or Proteome Database (.fasta)	MSGF+, X!Tandem, Comet	Not available	PGTools [180]
		MS/MS spectra (.mgf) Genome Database (.fasta)	ProteinPilot	https://proteogenomics.page.link/galaxyp	Galaxy-P [39]
		MS/MS spectra (.mgf) Peptide Database (.fasta) Genome coordinate (.gff3)	OMSSA, X!Tandem	https://proteogenomics.page.link/proanno	ProteoAnnotator [186]
		MS/MS spectra (.mzML) GenomeVarDB (.fasta) Annotation file(.gtf)	MSGF+	https://proteogenomics.page.link/ipaw	IPAW [13]
		MS/MS spectra (.mzXML) Proteome Database (.fasta)	JUMP	https://proteogenomics.page.link/jumpg	JUMPg [187]
		MS/MS spectra (.mgf) Metagenome (.fastg)	MSGF+	https://proteogenomics.page.link/g2pro	Graph2Pro Var2Pep [188]
		MS/MS spectra Transcriptome model (.gtf) Sequence database (.fasta)	MSGF+	https://proteogenomics.page.link/next	NextSearch [189]
		MS/MS spectra (.mzXML) Annotated neucleotide and proteins (gbk, fna, faa)	MSGF	https://proteogenomics.page.link/pgp	PGP [190]
	Multi-core	MS/MS spectra (.mgf, .dta, .pkl) Genome or Proteome Database (.fasta)	Built-in	https://proteogenomics.page.link/peppy	Peppy [8]
		MS/MS spectra(.mgf) Genome database (.fasta)	MSGF+, X!Tandem, OMSSA	https://proteogenomics.page.link/pgminer	PGMiner [191]
		MS/MS spectra (msfile) Genome database (.fasta)	X!Tandem, Mascot, MSGF+, IPeak, MyriMatch, OMSSA	https://proteogenomics.page.link/pga	PGA [192]
	Single-core	MS/MS spectra (.mgf) Genome database (.fasta)	MassWix, OMSSA, X!Tandem InsPecT	https://proteogenomics.page.link/genosuite	GenoSuite [193]
		MS/MS spectra (.mgf) Genome file divided into chromosomes (.fasta) RNA files (.bam/sam)	MSGF+ recommended	https://proteogenomics.page.link/enosi	Enosi [4]
		MS/MS spectra (.mgf) Protien and Genome database (.fasta)	External	https://proteogenomics.page.link/bpgpipeline	Bacterial Proteogenomic Pipeline [194]
		MS/MS spectra (.mgf) Transcriptome or Gene sequence (.fasta)	MSGF+	https://proteogenomics.page.link/mspro	MSProGene [195]

**TABLE 4.** Different visualizations available in proteogenomic tool.

Tool	Visualization
PGTools	Outputs BED file for Circos plots to visualize peptide loci in genomic context.
Galaxy-P	Visualizer for spectra and PSMs. Outputs GFF3 file for visualizing peptide mapping in a genome browser.
IPAW	In validation stage, plot of distribution for delta pi, precursor mass and match score are output.
ProteoAnnotator	Outputs GFF3 file for visualization in genome browser for top-scoring alternative loci.
GenoSuite	Outputs xml file for genomic context of each identified peptide. The xml file can be input to PSM plotter for visualization.
Bacterial Proteogenomic Pipeline	Outputs GFF3 file for visualization of spectral count for each type of experiment.
JUMPg	Outputs BED files containing information on the genomic locations of peptides which can be uploaded to UCSC genome browser for visualization.
PGMiner	Uses integrated genomic viewer for identified peptides.
NextSearch	Outputs GFF file for proteome-genomic/proteome-transcriptomic mapping for visualizing in a genome browser e.g. UCSC.
PGP	Outputs GFF3 files for visualizing annotations.
MSProGene	Outputs GTF file for visualizing the coordinates, confidence, and the number of spectrum matches.
PGA	Exports HTML report file for each PSM for visualized quality control.

## Footnotes

1 Wetterstrand KA. DNA Sequencing Costs: Data from the NHGRI Genome Sequencing Program (GSP) Available at www.genome.gov/sequencingcostsdata. Accessed [5/1/2019].

2 https://www.youtube.com/watch?v=fCd6B5HRaZ8

3 https://www.pacb.com/products-and-services/sequel-system/

4 https://nanoporetech.com/products/minion#

5 https://nanoporetech.com/products/comparison#platform&

6 *De novo* Genome Assembly for Illumina Data Available at: https://www.melbournebioinformatics.org.au/tutorials/tutorials/assembly/a-ssembly-protocol. Accessed [5/1/2019].
